# Scanning precession electron tomography for three-dimensional nanoscale orientation imaging and crystallographic analysis

**DOI:** 10.1038/ncomms8267

**Published:** 2015-06-01

**Authors:** Alexander S. Eggeman, Robert Krakow, Paul A. Midgley

**Affiliations:** 1 Department of Materials Science and Metallurgy, University of Cambridge, 27 Charles Babbage Road, Cambridge CB3 0FS, UK

## Abstract

Three-dimensional (3D) reconstructions from electron tomography provide important morphological, compositional, optical and electro-magnetic information across a wide range of materials and devices. Precession electron diffraction, in combination with scanning transmission electron microscopy, can be used to elucidate the local orientation of crystalline materials. Here we show, using the example of a Ni-base superalloy, that combining these techniques and extending them to three dimensions, to produce scanning precession electron tomography, enables the 3D orientation of nanoscale sub-volumes to be determined and provides a one-to-one correspondence between 3D real space and 3D reciprocal space for almost any polycrystalline or multi-phase material.

## Abstract (web summary)

High-resolution microscopy allows imaging of information on the atomic scale. Here, by combining precession electron diffraction with scanning transmission electron microscopy, the authors demonstrate an efficient, alternative technique to determine the three-dimensional orientation of materials.

## Graphical abstract

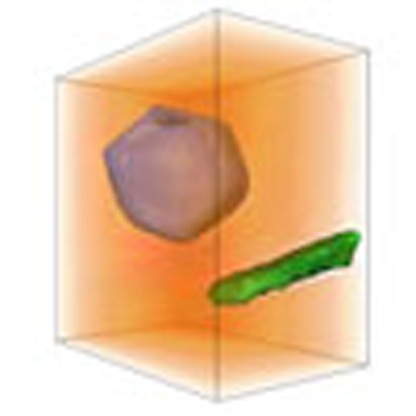

The morphological, composition and crystallographic complexity of modern materials and devices has driven the development of sophisticated microscopy tools to enable imaging and analysis across many length-scales. By combining scanning transmission electron microscope (STEM) imaging^1^ with spectroscopy using X-rays^2^ or energy loss^3^ and with tomographic acquisition^4^,^5^, multi-dimensional data sets^6^ provide unique spatially resolved physico-chemical information not achievable by any other method^7^. The local crystallography of materials may be studied using a number of techniques, including electron holography^8^ and lensless imaging/coherent diffraction^9^, which rely on a coherent source of electrons and phase retrieval algorithms, as well as more conventional dark-field approaches. Here though, we use a direct method for determining local orientation and crystallography, one that is amenable to being coupled with a STEM approach, namely precession electron diffraction (PED)^10^. In PED, a focused probe is rocked in a hollow cone about the optic axis above the specimen and de-rocked below, to produce a diffraction pattern of the same geometry as a conventional pattern but one which contains a larger number of reflections and where the diffracted intensities, by virtue of the rocking action of the Ewald sphere during the precession, are integrated across the Bragg condition. PED is primarily used for solving unknown structures^11^ but by rastering the beam across a 2D field of view, acquiring 2D PED patterns pixel by pixel, a four-dimensional (4D) data set can be acquired (a ‘diffraction-image'). The crystal orientation can be determined at each pixel in real space by comparison with previously simulated patterns residing in an on-line library^12^. Precessing the beam in such scanning experiments has been shown to improve the fidelity and reliability of two-dimensional (2D) orientation maps^13^ by averaging over small out-of-plane tilt variations and reducing the effects of dynamical diffraction associated with local thickness changes. Here we combine this scanning PED (or SPED) technique with electron tomography^14^ to determine the orientation of a sub-volume of the material of interest. For brevity, we refer to this technique as scanning precession electron tomography (SPET).

There are existing methods to enable 3D orientation mapping at a variety of length-scales. X-ray diffraction contrast tomography allows the orientation of grains in a 3D volume to be determined with sub-micron resolution^15^. In the scanning electron microscope (SEM), crystal orientation can be determined using electron back-scattered diffraction (EBSD) patterns. When combined with focused ion beam (FIB) milling it is possible to analyse the orientation slice-by-slice to yield 3D orientation information with a resolution of ∼(50 nm)^3^. This is clearly a destructive technique where further experiments on the volume of interest are impossible. Typical applications of this ‘dual-beam' approach are to the inter-grain orientation relationships in polycrystalline metals^16^, but deformation of micron-sized pillars, for example, has also been investigated^17^. In the TEM, by recording hollow-cone dark-field (HCDF) images as a function of the cone angle, azimuthal angle and the sample tilt, it is possible to reconstruct the 3D orientation of polycrystalline materials, in a manner analogous to that of the X-ray technique^18^. However, the acquisition time using the HCDF approach may be long and the corresponding electron dose may prohibit its use on beam-sensitive materials.

By contrast, the SPET method records PED patterns, pixel by pixel, tilt by tilt, analysing the data *post facto*, enabling the use of all scattered intensities in each diffraction pattern without destruction of the sample. As an example, we show here the 3D crystallographic analysis of a sample of a Ni-base superalloy ATI 718Plus^19^,^20^.

## Results

### Diffraction pattern analysis and decomposition

In simple terms, the microstructure of the alloy can be described as a Ni-rich f.c.c. matrix (γ-phase) strengthened with γ' precipitates (with approximate composition Ni_3_Al) and a variety of other inter- and intra-granular precipitates. In the volume studied here, we focus on the matrix, an inter-granular Nb-rich carbide and a lath-like precipitate, the hexagonal η-phase with approximate composition Ni_6_Nb(Al,Ti). The fine-scale γ' precipitates, coherent with the matrix, are not studied here.

**Figure 1:**
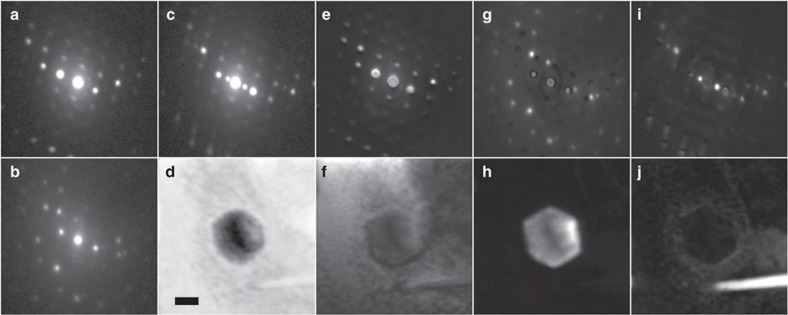
SPED data from the nickel superalloy sample. Representative PED patterns corresponding to (**a**) the matrix phase (γ), (**b**) a metal carbide precipitate and (**c**) a η-phase lath-like precipitate. (**d**) A virtual bright-field image of the region with the carbide at the centre and the η-phase at the bottom right. (**e**,**f**) show the NMF-decomposed component pattern and loading map representative of the γ-matrix adjacent to the carbide. The component pattern and loading maps, representative of the carbide particle and η-phase, are shown in (**g**,**h**) and (**i**,**j**), respectively. Scale bar, 100 nm.

A tilt series of SPED ‘diffraction-images' was acquired from the volume of interest—details are given in the Methods section. Figure 1 shows representative PED patterns acquired at zero sample tilt from regions dominated by (a) matrix, (b) carbide, (c) η-phase. Figure 1d shows a virtual bright field (VBF) image formed by plotting, pixel by pixel, the recorded intensity of the undiffracted beam; a large facetted precipitate is seen near the centre.

Looking carefully at the diffraction pattern in Fig. 1b it is evident that the pattern is actually composed of reflections from more than one phase. The sample thickness is sufficiently large that the carbide and the η phase are partially or completely surrounded by the matrix. For any complex material composed of nanoscale grains and/or precipitates, this will be a common situation.

Thus, in general, we need a robust, unbiased method to separate the diffracted intensities for each phase from the ‘mixed' PED pattern. This example is a particularly difficult case because of the coherency of the secondary phases to the matrix, and the similarity of the planar spacings of the phases involved; in terms of the diffraction pattern, they share common g-vectors.

To decompose each diffraction pattern into the constituent components from each phase (or orientation), we use a form of multivariate analysis^21^ called non-negative matrix factorization (NMF)^22^ implemented within the Hyperspy package [ http://www.hyperspy.org/], which has proved valuable for a variety of image and spectral processing. Further details are given in Methods but, briefly, NMF decomposes each PED pattern into a linear sum of component patterns (basis functions) each weighted by a loading (coefficient). The number of component patterns is limited ideally to the number of phases (or orientations) in the volume studied. Importantly, unlike, for example, principal component analysis (PCA), both the component patterns and the loadings are positive, enabling easier physical interpretation and their use directly in understanding the 3D crystallography of the volume.

In this example, we find that for each SPED ‘pattern-image', composed of 14,400 PED patterns, 20 component patterns provide an optimum fit, 17 of which describe the different orientations of the matrix, which varies relatively slowly across the field of view, one is related to the carbide and one to the η-phase.

In Fig. 1e,f we show the component pattern and loading map associated with the γ matrix, those for the facetted carbide particle are shown in Fig. 1 g,h and those for the η-phase are shown in Fig. 1i,j. The loadings associated with the latter two components confirm the spatial origins of those phases: where the loading is brightest indicates where the corresponding component pattern is most dominant.

No decomposition of this sort is perfect (or unique) and artefacts can be seen in the individual component patterns, typically as weak annuli of intensity. However, this does not preclude the use of component patterns for orientation determination, treating them as true diffraction patterns.

### Reconstruction of real space and reciprocal space volumes

The NMF-based decomposition was repeated at each tilt and the component patterns and loadings are shown in Supplementary Fig. 1. Thus, the decomposed SPET data set provides 3D real space morphological information in the form of the tilt series of loadings in addition to the 3D reciprocal space information in the form of template-matched component patterns.

The tilt series of loadings can be reconstructed using a geometric tomography, or ‘shape-from silhouette', reconstruction algorithm^23^, which returns a 3D ‘envelope' within which the full 3D orientation is known from the reconstructed reciprocal lattice^24^,^25^ from the complementary tilt series of component patterns. Thus, there is a one-to-one correspondence between the 3D volume reconstructed in real space and the 3D orientation within that volume.

**Figure 2:**
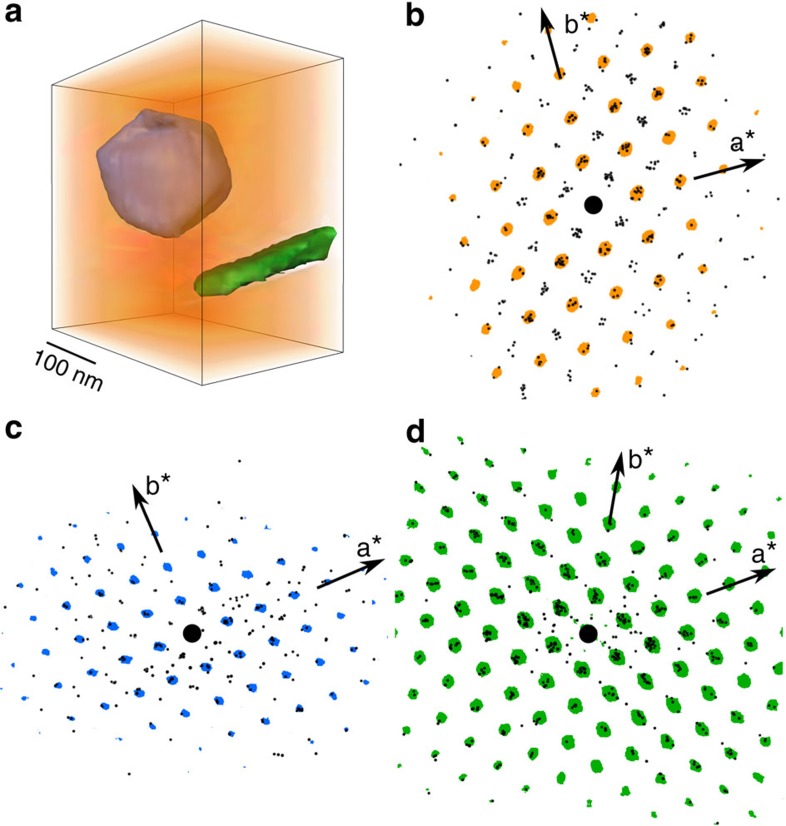
Real space and reciprocal space reconstructions of the Ni-base superalloy microstructure. (**a**) 3D reconstruction of the superalloy volume showing the faceted metal carbide (blue), the η-phase (green) and the surrounding matrix phase (orange). Scale bar, 100 nm. [001] zone axis projections of the reconstructed 3D reciprocal spaces, corresponding to the three regions in **a**, are shown in (**b**) the matrix, (**c**) the metal carbide and (**d**) the η-phase. The coloured overlay of spots is the auto-correlation of the zero order Laue zone reflections extracted from the 3D reciprocal space reconstructions, with reciprocal lattice basis vectors clearly marked.

Figure 2a shows a 3D real space reconstruction of the phases in a volume approximately 0.5 μm × 0.5 μm × 0.2 μm in size. Figure 2b–d shows [001] projections of the reconstructed reciprocal lattice from matrix, carbide and η phase, respectively. The black dots represent the reflections identified in each component pattern through the tilt series. The deviation of the dots about their mean reciprocal lattice position arises from a number of experimental factors commonly encountered in 3D electron crystallography experiments. First, imperfections in the tilt goniometer can lead to errors in the recorded tilt value. Second, the broadening of reflections because of the finite thickness of the specimen (‘rel-rods') coupled with the curvature of the Ewald sphere can lead to a difference between the measured g-vector and the true reciprocal lattice point. Third, geometric distortions of the patterns due to imperfect projector lenses may lead to deviations from the correct position, especially for higher order reflections. Last, if there is any misalignment of the pattern centres during reconstruction then further displacement of the dots can occur.

To minimize the effects of the position errors, we use a difference vector analysis (essentially an autocorrelation) to improve the statistics of major clusters corresponding to the Bragg reflections^25^. In this way outlying points are reduced in significance and have less influence on the determination of crystal parameters.

In Fig. 2, the autocorrelation of the zero-order Laue zone (ZOLZ) reflections, that is, reflections in the plane normal to the zone axis viewing direction including the direct beam, are shown as coloured dots overlaying the projection of the reciprocal lattice reconstruction. The Bravais lattice of the matrix and carbide, in both cases cubic F, ensures that only reflections with all indices either odd or even are allowed. The two zero layer basis vectors in the patterns of both Fig. 2b,c correspond to [200]* and [020]*. As the pattern is a projection of a 3D reciprocal lattice, reflections in higher order Laue zones, for example 111, are also seen, projected to the centres of the square array of coloured dots.

From the reconstructed reciprocal lattices (shown in Supplementary Fig. 2), it was possible to determine the lattice parameters of the matrix, measured to be a=3.60 Å±0.06 Å, the carbide, a=4.43 Å±0.07 Å and the η phase, a=5.30 Å±0.06 Å, c=8.30 Å±0.08 Å, all in good agreement with the expected values. The carbide structure and lattice parameter is consistent with that of a Nb-rich carbide, supported by STEM energy-dispersive X-ray spectroscopy experiments (see Supplementary Fig. 3).

### Orientation and interface analysis

**Figure 3:**
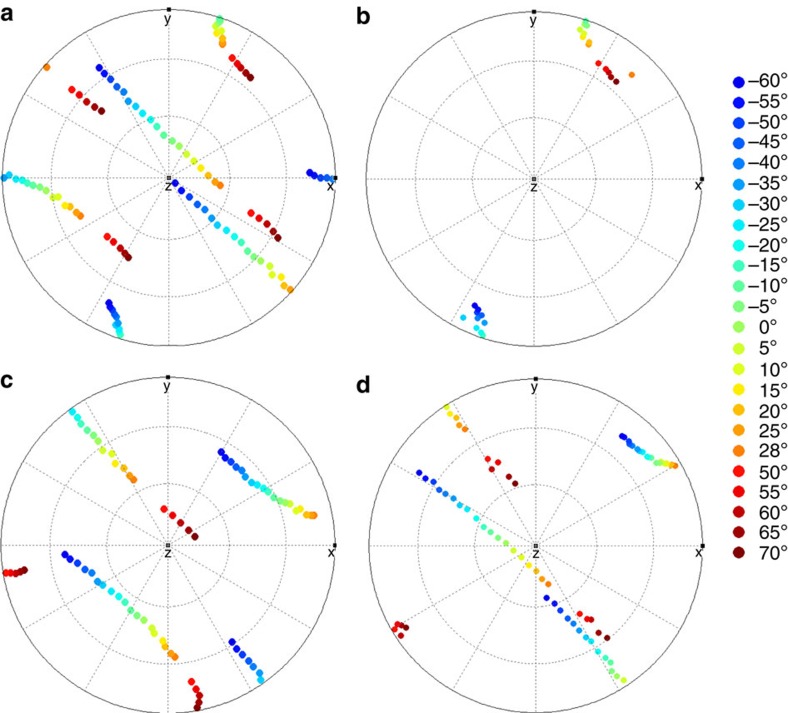
Pole figures showing the orientation of the phases in the tilt-series. (**a**,**b**) show pole-figures representing the {111} zone axes of the γ-matrix and the [0001] zone axis of the η-phase, respectively. The orientation at each step of the tilt series is plotted from red (+70°) to blue (−60°), with the tilt axis running approximately SW to NE. The orientation relationship between these phases can be gauged from the matching poles. (**c**,**d**) show the {100} pole figures for the matrix and the metal carbide, respectively.

Individual component patterns from the tilt-series were indexed, using the NanoMegas ASTAR ‘Index' program (examples from the matrix and carbide phases are shown in Supplementary Fig. 4), to provide complete 3D orientation information for the observed phases. The resulting orientation data were analysed using the MTEX Matlab toolbox^26^ to provide pole-figures for the three phases; examples are shown in Fig. 3. The known orientation relationship between the γ- and η-phases, {111}γ//(001)η, provides confirmation of the successful analysis: there is a clear match between one set of {111}γ poles, shown in Fig. 3a and the (001)η poles in Fig. 3b. It is worth noting that the η-phase has a lath-like morphology, with the coherent interface corresponding to the larger flattened surfaces (minimizing the interface energy between the two phases during growth).

**Figure 4:**
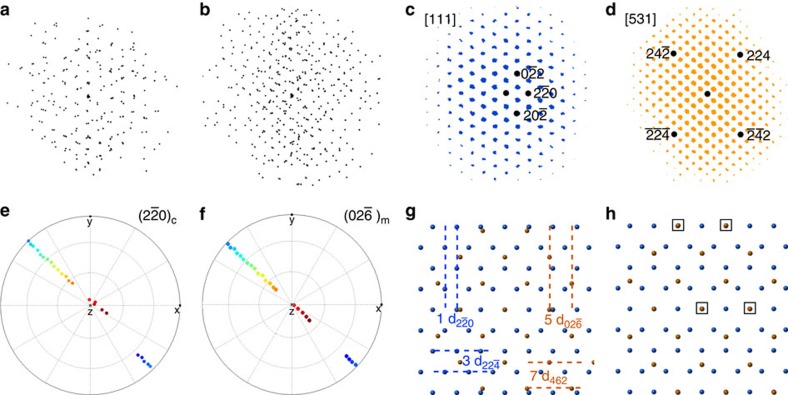
Orientation and structural information from the interface between the matrix and the metal carbide particle. (**a**,**b**) show the 3D reciprocal lattice reconstruction for the metal carbide and matrix, respectively, projected parallel to the normal of the large facet of the carbide particle, approximately the orientation seen in Fig. 2a. (**c**,**d**) show the corresponding 2D autocorrelations of these two projections, to highlight the periodicity and enable easier indexing. A pole figure for the metal carbide (
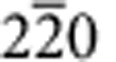
) planes is shown in (**e**) and the (
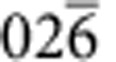
) planes of the matrix γ-phase is shown in (**f**). A schematic representation is shown in (**g**) of the final atomic layer in the carbide (111) plane in blue and the first layer of the (
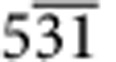
) plane of the γ-phase in orange, along with the traces of planes indicative of the interface relationship. In (**h**) the atomic positions of the γ-phase have been relaxed to accommodate a biaxial strain of 7.5% in the [
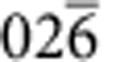
] direction and 5% in the [462] direction. The squares indicate part of a coincident site lattice (CSL) at the matrix/carbide interface.

A similar comparison between low index poles in the carbide and the matrix shows no obvious crystallographic registry, the example of the {100} poles is shown in Fig. 3c,d. To determine the carbide/matrix orientation relationship, we rotated the complete data set such that the 3D reciprocal lattice of the carbide and the matrix could be viewed parallel to the normal to the large facets of the carbide particle, see Fig. 4a,b. The geometry of the projected reciprocal lattices becomes clearer after performing an autocorrelation, see Fig. 4c,d. Now the patterns can be indexed and it becomes evident that the facet normal is parallel to the [111] zone axis of the carbide and the [
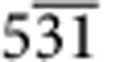
] zone axis of the matrix. Further, the mutually perpendicular (
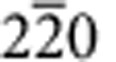
) and (
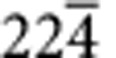
) planes of the carbide, lying in the [111] zone, are parallel to the (
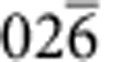
) and (462) planes of the matrix, lying in the [
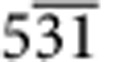
] zone. We can illustrate this relationship further using pole figures as before, see Fig. 4e,f, which show the coherency between a subset of {220} planes in the carbide and {026} planes in the matrix.

This orientation relationship, to our knowledge not previously reported for f.c.c. systems, can be illustrated schematically in real space, as seen in Fig. 4g,h that overlays the last atom layer of the matrix with the first atom layer of the carbide. The biaxial mismatch strain is 5%, between the (
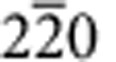
) carbide and the (
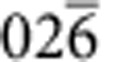
) matrix planes and 7.5% for the (224) carbide and the (462) matrix planes. The mismatch would be rather large to accommodate coherently at a simple planar interface. Misfit dislocations are possible but the {531} surface is unusual in that it is chiral and can be considered as a kink-step {111} vicinal surface^27^,^28^ so that a component of the strain could be distributed both within the {111} terraces and at each kink. Analysis of a matrix component pattern (shown in Supplementary Fig. 5), whose loading is strongest in a region surrounding the carbide, indicates a tensile strain in the matrix consistent with that needed to accommodate the carbide/matrix misfit.

The orientation relationship between the matrix and the carbide particle provides some insight into the evolution of the superalloy microstructure. The formation temperature of the carbide is ∼1,200 °C and will form during or just after solidification from the melt^29^. The closed-packed nature of the {111} planes of the carbide structure provides the basis for the development of large {111} facets seen here, minimizing the energy of formation. In our case, the sample has undergone hot working (forging), which almost inevitably leads to recovery and recrystallization of the work-hardened structure. The relatively low interface energy between the near-coherent carbide {111} and matrix {531} surfaces suggests that the carbide likely acted as a heterogeneous nucleation site for a new sub-grain of the matrix during recrystallization. Other carbides (and carbo-nitrides) with similar lattice parameters found in many superalloy systems may also demonstrate similar orientation relationships with the matrix.

## Discussion

The successful development and application of this new 3D tomographic technique is based on the ability to separate component patterns from a ‘mixed' set of data. Such decomposition, and indeed data reduction in general, is becoming a key feature of many analytical techniques where ‘multi-dimensional' data sets are acquired. MSA offers a flexible means to achieve this. In electron microscopy, while MSA is growing in popularity for electron spectroscopy (EDX and EELS), its use to date for series of images or diffraction patterns has been very limited.

Here we have used NMF to derive component patterns and associated loadings, which, in part by virtue of the positivity constraint inherent in NMF, can be interpreted as resembling phase-specific diffraction patterns and (dark-field) images. However, it is important to note that, as with any MSA method, NMF simply decomposes the data into statistically significant functions and associated weightings. Any decomposition of this type does not provide a unique answer but has the great advantage of massive data reduction, is relatively free of user bias and can, in principle, be automated.

The applicability of this approach to SPET data is confirmed by the successful reconstruction and ‘segmentation' of the reciprocal lattice geometry of the three phases seen here. The loadings indicate strong localization in real space and this allows clearly defined real space reconstructions of the three phase volumes.

In our experiments, we use only a modest precession angle (to preserve high spatial resolution, see Methods), but is sufficient to expand the range of reciprocal space covered by the precessing Ewald sphere and enable reliable pattern matching and accurate lattice parameter measurement.

Understanding the properties of polycrystalline and multi-phase materials requires detailed knowledge of the microstructure and especially the orientation relationships between neighbouring grains and regions of material. By exploiting the one-to-one correspondence in SPET between 3D real and reciprocal space information, we have revealed a new interface relationship that provides insight into the thermo-mechanical history of a Ni-base superalloy. Moreover, SPET now offers a means to study 3D orientation relationships at the nanoscale across a wide range of microstructures and materials, including nanocrystalline metals 30, order-disorder relationships or domain structures^31^ and 3D strains in, for example, semiconductor device structures^32^. With the advent of ultra-fast and sensitive cameras with direct electron detection, there is the possibility to extend this technique to beam-sensitive material such as organic, pharamaceutical and biological crystals^33^,^34^.

## Methods

### Electron microscopy experiments

SPED was performed on a Philips CM300 FEGTEM operating at 300 kV. The scan and precession were controlled through a Nanomegas Digistar connected to the microscope and running the ASTAR software package. For the experiments, a small-angle convergent probe (convergence semiangle of 1.4 mrad) was used to allow the separation of closely spaced reflections (expected for the η-phase). Source size demagnification (first condenser lens strength) was modest to retain a high beam current to facilitate short exposure times at the expense of a larger probe size. A relatively small precession angle of 0.5° (8.7 mrad) was chosen to increase sufficiently the number of reflections detected in each diffraction pattern, and hence increase the reliability of indexing, but not to lead to excessive beam-broadening through the specimen volume^10^. We estimate the final probe size was 5 nm in diameter.

Each scan was 120 × 120 pixels^2^ with pixel steps of 7.5 nm and a PED pattern recorded at each point, patterns were recorded using a Stingray CCD camera, which captures the image on the binocular viewing screen. The recorded image was corrected for geometric distortion before crystallographic analysis. The precession frequency was 100 Hz and the dwell time at each pixel was 20 ms to ensure complete integration around the precession circle.

The sample was tilted at 5° intervals from −60° to +70° using a Fischione 2020 tomography holder.

### Statistical decomposition

Non-negative matrix factorization (NMF) was performed using the ‘Hyperspy' software package. Each SPED data set was loaded as a stack of 14,400 diffraction patterns (from the 120 × 120 pixel^2^ scan). NMF decomposition of the image stack was performed assuming Poisson noise and for a range of expected components. Through trial-and-error, a total of 20 components was found to return results that reliably matched features we were able to identify for each of the major phases (matrix, carbide and η-phase) in the sample while minimizing the calculation time. Examples are shown in Fig. 1.

NMF constrains both the components and the loadings to be positive, as a reasonable *a priori* constraint and in the hope that both may then be physically interpretable: the component patterns resemble conventional diffraction patterns and the loading maps virtual dark-field (VDF) images. By decomposing the data using NMF, the component patterns can be used directly as input for the reciprocal lattice reconstruction and the loadings as images for real space reconstruction.

As an example, the component patterns and loading maps, associated with the facetted metal carbide particle across the tilt series, are shown in Supplementary Fig. 1. The loading maps have been binarized to simplify the image input for the geometric tomographic reconstruction of the carbide particle—to produce a 3D ‘envelope'. The results from +30° to +45° were corrupted by shadowing from a piece of carbon film and are not used in the analysis.

A second component pattern/loading pair was associated with the carbide, another with the η phase and the remaining 17 component/loading pairs associated with the matrix. The large number reflects the complex orientation changes seen in the matrix brought about by slowly varying tilt, bending and the presence of a low angle boundary within the field of view. Although not of importance here, this indicates the sensitivity of the NMF routine in identifying small orientation changes in strained material.

### Tomography reconstructions

Reconstruction of the real-space volumes from the loading maps of the three phases was undertaken using FEI Inspect3D software. Volume rendering was achieved using the Avizo Fire software package. Reconstruction of the reciprocal lattice for each component was performed using in-house software.

### Crystal structure determination

The f.c.c. crystal structure of the matrix was confirmed by analysis of the <001> and <011> projections of the reconstructed 3D reciprocal lattice, as shown in Supplementary Fig. 2a and b, respectively. The zero-order Laue zone (ZOLZ) reflections were identified by analysing a central slice (containing the origin) of the reconstruction normal to the viewing direction. As the tilt series is not complete (missing wedge of information), the ZOLZ was only partially sampled in the central slice. However, by using auto-correlation, the ZOLZ geometry was improved greatly, see coloured overlay of spots.

From these two projections, a body-centred reciprocal lattice is evident, confirming an f.c.c. real-space lattice. Using the internal ASTAR calibration values (0.604 mrad per pixel, an effective camera length of 126mm), the lattice parameter for the matrix was determined to be 3.60 Å+/−0.06 Å, which agrees well with the expected value for the γ-phase in a nickel-base superalloy^29^. The error was estimated by considering the variation in orientation of the reciprocal lattice. Given the possibility of up to 0.5° error in the tilt axis measurement, combined (in quadrature) with the cluster size of ∼0.2° around each reciprocal lattice point, the aligned sample was mis-tilted by 0.54° in eight randomly determined directions. Each of the eight reconstructed reciprocal lattices underwent the same autocorrelation and geometric analysis to determine the cell parameters. The mean and s.d. of these parameters are the reported values.

In the same way, the reconstructed 3D reciprocal lattice of the metal carbide was oriented along the <001> and <011> zone axes, as shown in Supplementary Fig. 2c and d, respectively. Although the number of reflections uniquely assigned to the carbide is fewer than that of the matrix, with a somewhat noisier reconstruction as a result, the figures still clearly show reciprocal space projections consistent with a f.c.c structure. The measured lattice parameter of 4.43+/−0.07 Å is consistent with (Nb/Ti)C^29^, with the NaCl structure, known to exist in this superalloy. Ti and Nb are both strong carbide forming elements in this alloy system and the corresponding carbide lattice parameters vary, depending on the non-stoichiometry of the carbon, between 4.33 Å and 4.27 Å for TiC and 4.47 Å and 4.42 Å for NbC, respectively.

### Composition of the metal carbide

Chemical mapping of the region of interest was performed on an FEI Tecnai Osiris (S)TEM, fitted with a large solid-angle silicon drift detector (SDD) for STEM-EDX measurements. A summary of the results are shown in Supplementary Fig. 3. The upper half of the figure shows element maps formed from the K peaks of the expected alloy elements, the lower half shows the integrated EDX spectrum for the central portion of the facetted particle. The Nb signal is clearly strongest in this region and, together with the large Nb:Ti ratio in the EDX spectrum, confirms the particle is Nb-rich.

### Crystal orientation determination

The structure analysis was confirmed by inspection of the output from pattern fitting (software provided as part of the ASTAR system) between structure libraries (generated with the measured lattice parameters listed above) and the component patterns returned by the decomposition. The comparison of the component pattern (P) with the template library of patterns (T) is quantified using a Correlation Index (Q) 35:


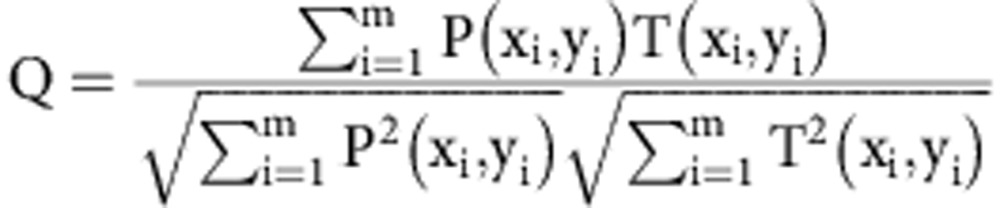


Supplementary Fig. 4a shows and example of a match between the best fit in the pattern library (as a coloured overlay) for the component pattern corresponding to the matrix at a tilt of −5°. The orientation is confirmed in Supplementary Fig. 4b that shows the Correlation Index for all of the unique patterns in the library as a function of their position in the stereographic triangle (a symmetry independent sector of reciprocal space for the f.c.c. crystal). For the figure, the lower the value of Q the poorer the match and this is assigned a light tone, the higher values of Q (better fits) are assigned a darker tone; the position of the best fit is indicated with a coloured dot. The same analysis is shown for the carbide component in Supplementary Fig. 4c and 4d, respectively. The stereographic pole figures were produced using the MTEX package for MATLAB.

### Matrix strain and composition near the carbide

The elemental maps in Supplementary Fig. 4 show the presence of the fine-scale γ' particles, rich in nickel and aluminium/titanium (γ' is based around the ordered Ni_3_Ti or Ni_3_Al structure) and the corresponding absence of the other elements (iron, chromium and cobalt). These other elements, together with oxygen, are present throughout the matrix but appear to be enhanced in the region of the carbide.

One of the 17 component patterns associated with the matrix is shown in Supplementary Fig. 5a, together with its corresponding loading in 5b. Although at first glance the loading map appears to be indicating the carbide, the component pattern can be indexed only as one associated with the matrix phase. Thus, the loading map must be associated with a region of matrix above and/or below the carbide. Moreover, the strong contrast seen in the loading map matches the EDX elemental map contrast for all the elements except Ni and Ti (which are preferentially in the carbide). The best match for the component pattern in Supplementary Fig. 5a is, however, found when the matrix is strained isotropically in a tensile manner by 3.1%, qualitatively consistent with the strain needed to achieve the CSL seen in Fig. 4. The heterogeneous solute segregation in the matrix near the carbide, identified in the EDX elemental maps, may also be driven by strain at the carbide/matrix interface.

### Data availability

All relevant data present in this publication can be accessed at http://www.repository.cam.ac.uk/handle/1810/247679.

## Additional information

**How to cite this article**: Eggeman, A.S. *et al.* Scanning precession electron tomography for three-dimensional nanoscale orientation imaging and crystallographic analysis. *Nat. Commun.* 6:7267 doi: 10.1038/ncomms8267 (2015).

## Supplementary Material

Supplementary Information Supplementary Figures 1-5

Supplementary Movie 1 This movie shows the rotation of the volume reconstruction of the carbide particle (left) and the reciprocal lattice reconstructions of the carbide (centre) and the matrix (right). The initial position of all three reconstructions is set to the 0° tilt position in the tilt-series and in all cases the tilt-axis is oriented vertically. An indexed autocorrelation of the reciprocal lattice is overlaid to show the orientation of each phase.
