# Supplementary material for: Scanning precession electron tomography for three-dimensional nanoscale orientation imaging and crystallographic analysis
Source: Nat Commun. 2015 Jun 1;6:7267. doi: 10.1038/ncomms8267 (PMC4458861; doi:10.1038/ncomms8267)
Supplement: Supplementary Information — Supplementary Figures 1-5 [file ncomms8267-s1.pdf]

Supplementary Information

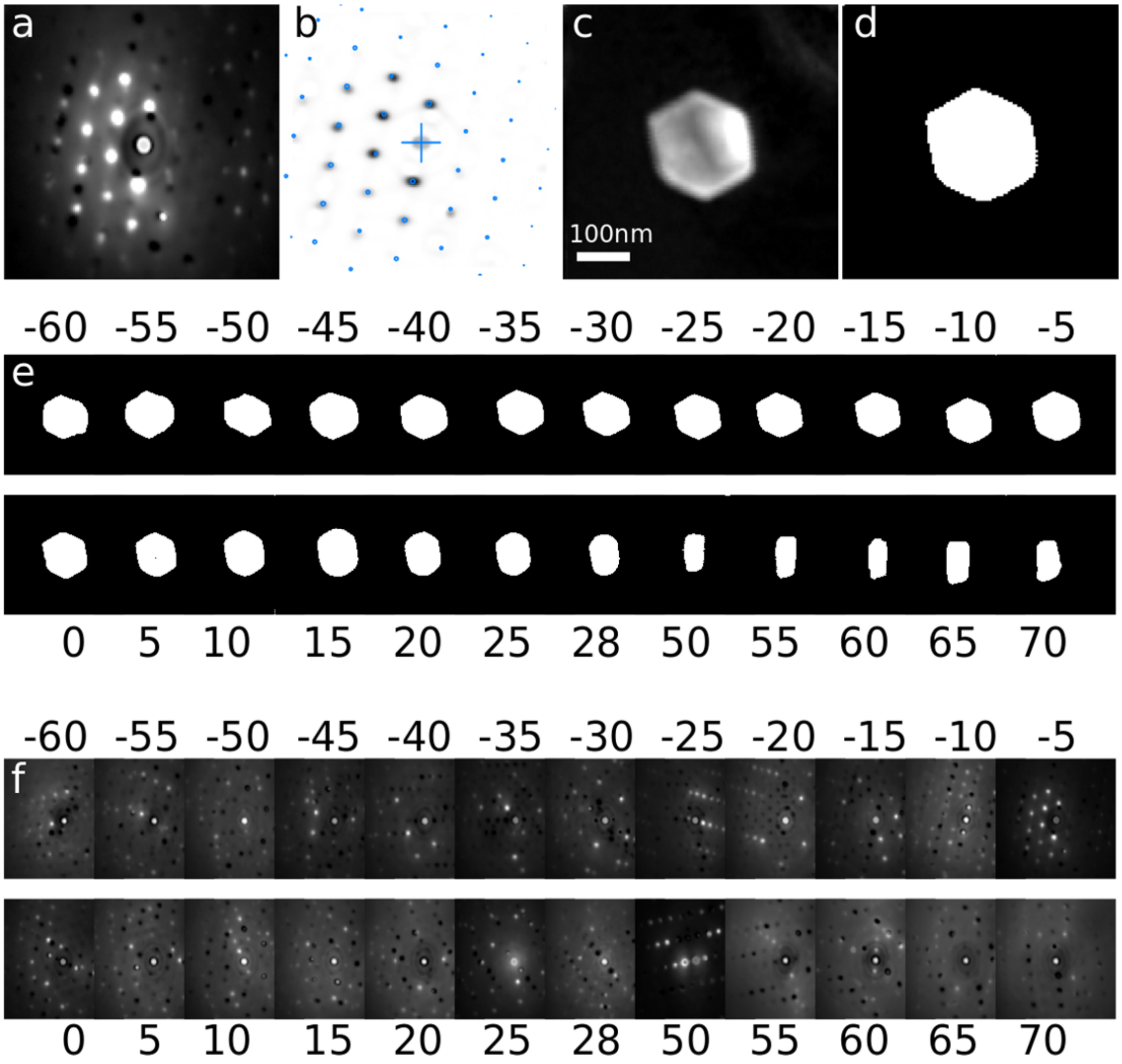

Supplementary Figure 1. NMF Decomposition results from the SPET tilt-series: (a) shows the component pattern associated with the carbide particle at -5° tilt, (b) shows the corresponding pattern match (after distortion correction) for orientation determination. (c) The component loading map and (d) a binary version of this loading map. (e) and (f) show the complete binary loading information and the component patterns for the carbide particle through the entire tilt-series. The numbers indicate the tilt angle.

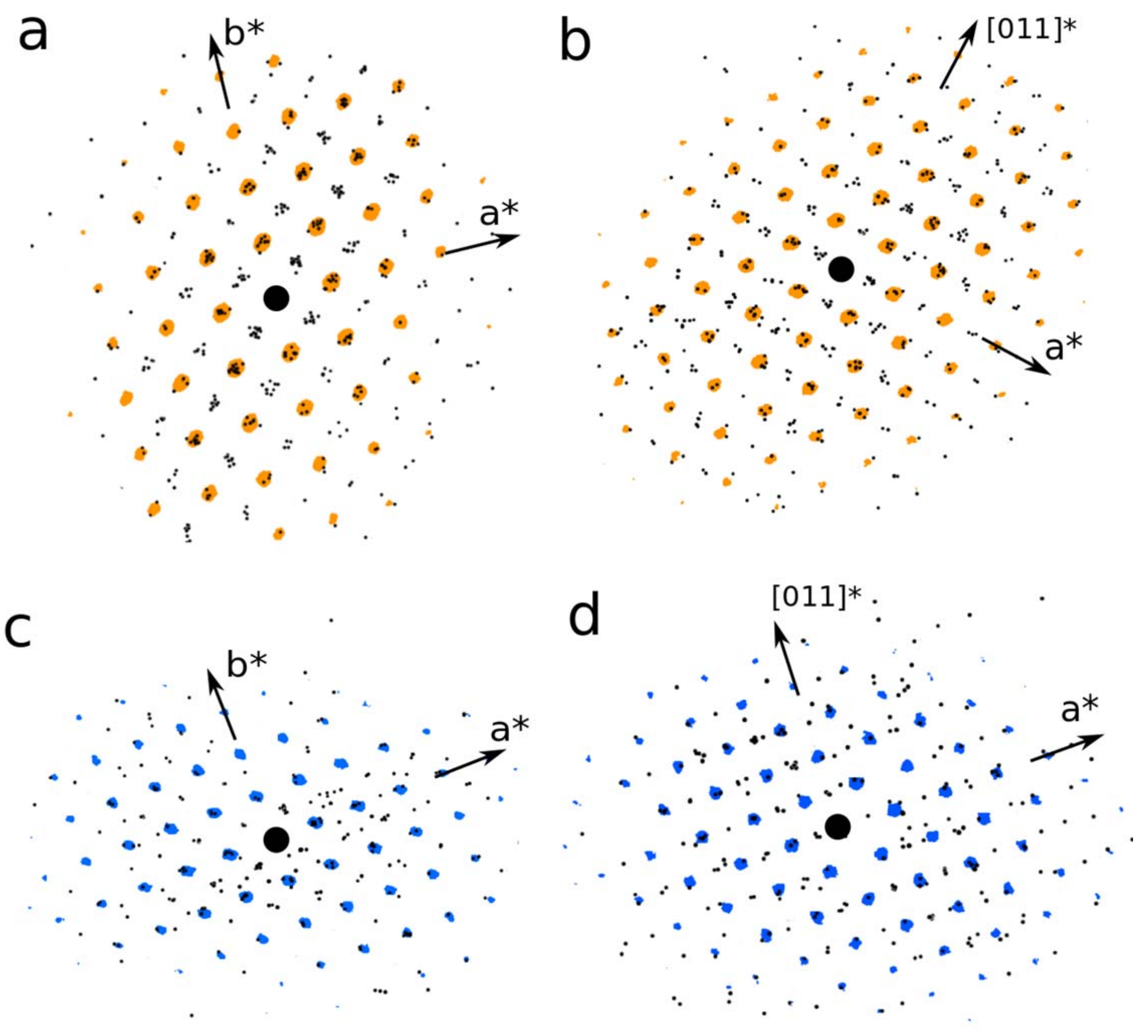

Supplementary Figure 2. Reciprocal lattice reconstructions. (a) and (b) show the reciprocal lattice of the matrix phase oriented parallel to [001] and [$01\bar{1}$] respectively. (c) and (d) show the reciprocal lattice for the carbide phase oriented parallel to [001] and [$01\bar{1}$] respectively. Autocorrelation of the ZOLZ reflections is added as coloured regions to highlight the geometry of the reciprocal lattice.

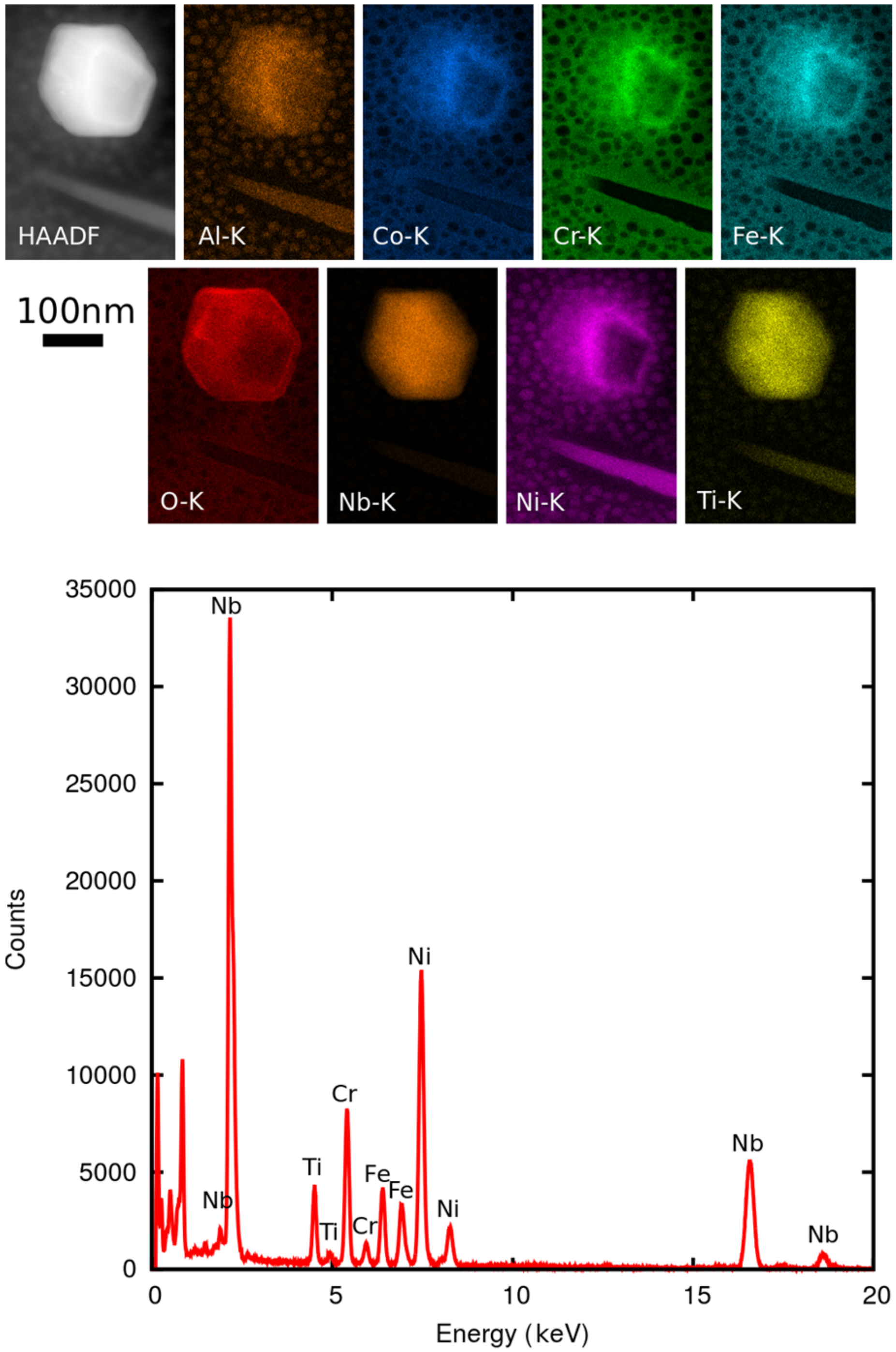

Supplementary Figure 3. . STEM-EDX results from the region of interest, the images show K-peak maps for the alloying elements in the system, a common scale bar for all of the images is included. The graph shows an integrated spectrum for the central portion of the carbide particle

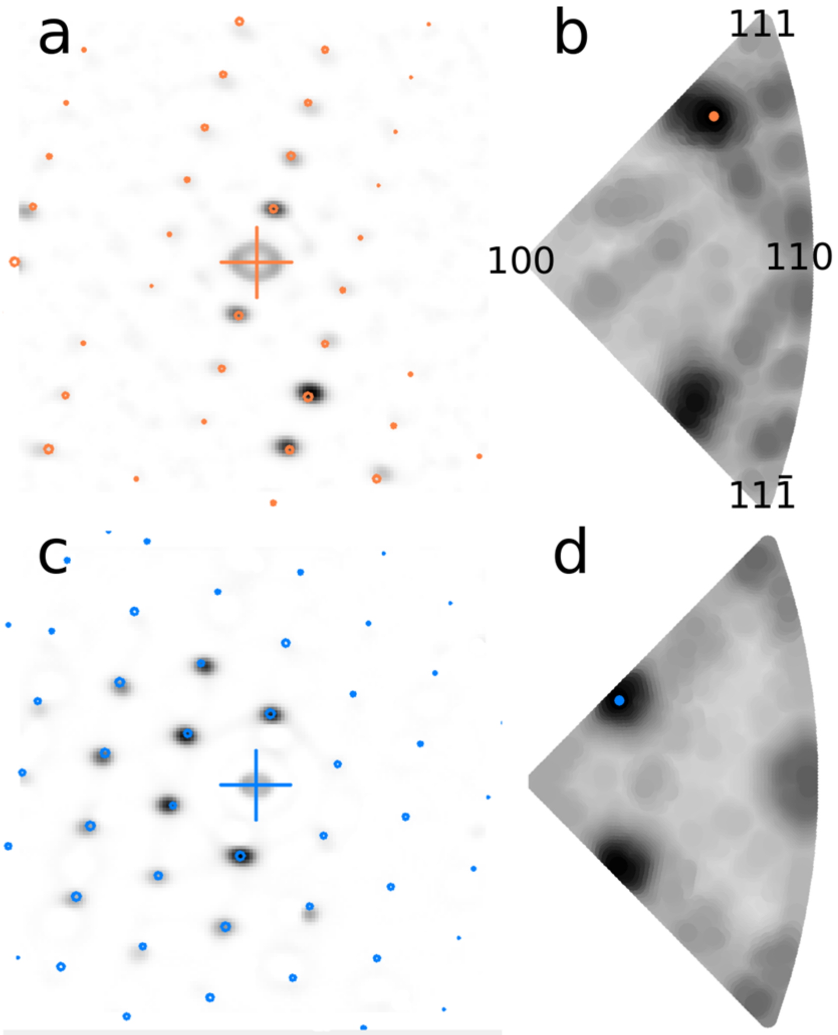

Supplementary Figure 4 Pattern matching (Correlation Index) results for the -5° tilt step of the SPET data. (a) the match between the matrix component pattern and the template library (coloured), the orientation of this pattern in the stereogram is shown in (b). (c) and (d) show the comparable pattern matching result for the component pattern corresponding to the carbide phase.

e

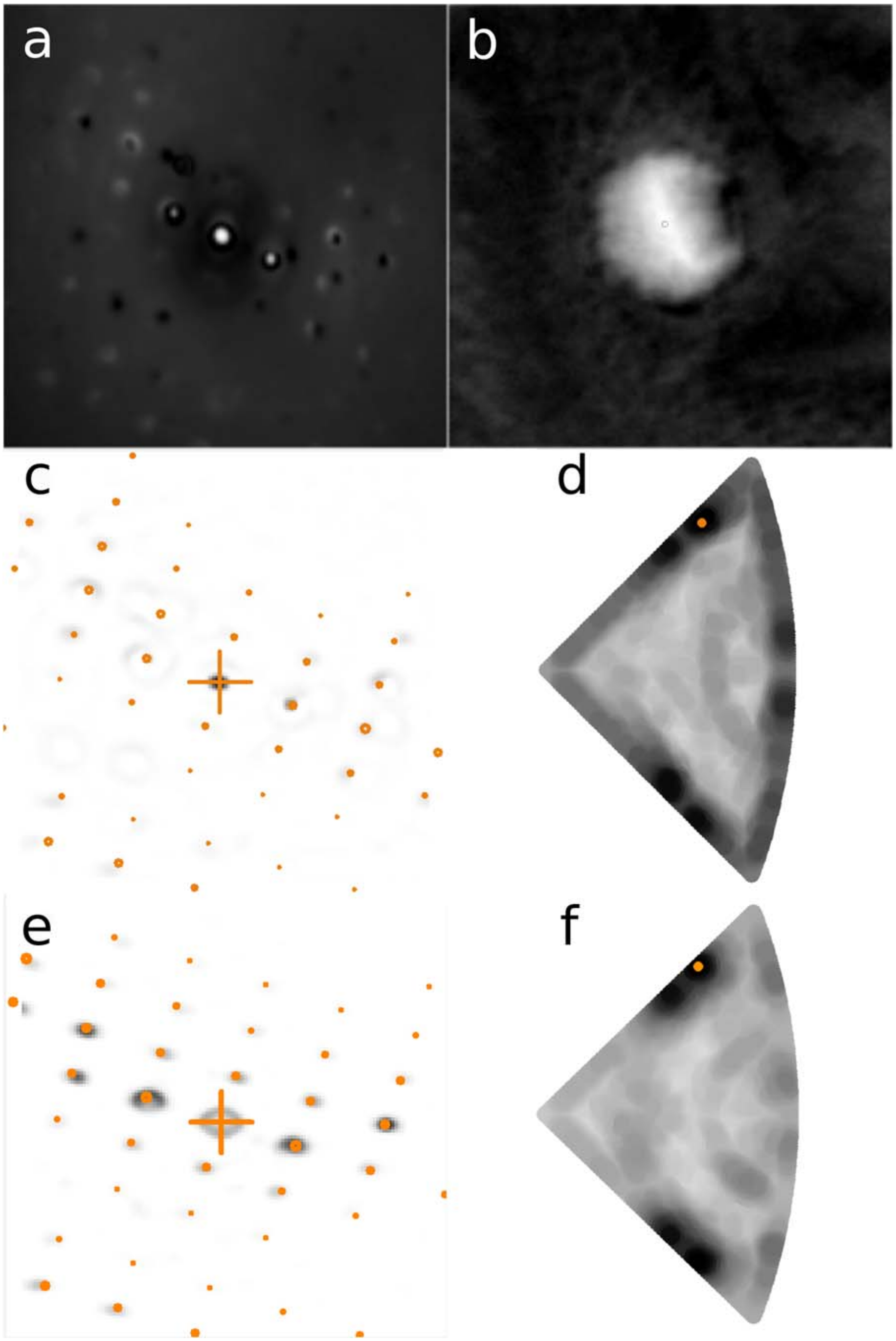

Supplementary Figure 5. (a) Component pattern and (b) loading map for a matrix phase component highlighting the region adjacent to the carbide. (c) Pattern match between the component map and a template pattern created by straining the matrix by 3.1%. (d) The orientation of the matching pattern in a stereogram. (e) and (f) show the pattern match and orientation for the un-strained matrix component away from the carbide particle; note the similar pattern and orientation compared to (c) and (d).
